# Evolution and Stress Responses of *CLO* Genes and Potential Function of the *GhCLO06* Gene in Salt Resistance of *Cotton*

**DOI:** 10.3389/fpls.2021.801239

**Published:** 2022-01-17

**Authors:** Xiaokang Fu, Yonglin Yang, Meng Kang, Hengling Wei, Boying Lian, Baoquan Wang, Liang Ma, Pengbo Hao, Jianhua Lu, Shuxun Yu, Hantao Wang

**Affiliations:** ^1^State Key Laboratory of Cotton Biology, Institute of Cotton Research, Chinese Academy of Agricultural Sciences (CAAS), Anyang, China; ^2^Shihezi Academy of Agricultural Sciences, Shihezi, China

**Keywords:** cotton, caleosin protein, salt tolerance, abscisic acid, *GhCLO06*

## Abstract

The caleosin (*CLO*) protein family displays calcium-binding properties and plays an important role in the abiotic stress response. Here, a total of 107 *CLO* genes were identified in 15 plant species, while no *CLO* genes were detected in two green algal species. Evolutionary analysis revealed that the *CLO* gene family may have evolved mainly in terrestrial plants and that biological functional differentiation between species and functional expansion within species have occurred. Of these, 56 *CLO* genes were identified in four cotton species. Collinearity analysis showed that *CLO* gene family expansion mainly occurred through segmental duplication and whole-genome duplication in cotton. Sequence alignment and phylogenetic analysis showed that the *CLO* proteins of the four cotton species were mainly divided into two types: H-caleosins (class I) and L-caleosins (class II). *Cis*-acting element analysis and quantitative RT–PCR (qRT–PCR) suggested that *GhCLOs* might be regulated by abscisic acid (ABA) and methyl jasmonate (MeJA). Moreover, transcriptome data and qRT–PCR results revealed that *GhCLO* genes responded to salt and drought stresses. Under salt stress, gene-silenced plants (TRV: *GhCLO06*) showed obvious yellowing and wilting, higher malondialdehyde (MDA) content accumulation, and significantly lower activities of superoxide dismutase (SOD) and peroxidase (POD), indicating that *GhCLO06* plays a positive regulatory role in cotton salt tolerance. In gene-silenced plants (TRV: *GhCLO06*), ABA-related genes (*GhABF2*, *GhABI5*, and *GhNAC4*) were significantly upregulated after salt stress, suggesting that the regulation of salt tolerance may be related to the ABA signaling pathway. This research provides an important reference for further understanding and analyzing the molecular regulatory mechanism of *CLOs* for salt tolerance.

## Introduction

Caleosins (*CLOs*) are calcium-binding proteins encoded by small gene families, sometimes called peroxygenases (*PXGs*) in databases, and are widely distributed in terrestrial plants (Khalil et al., 2014; Shen et al., 2016; Rahman et al., 2018). Caleosins (Pfam PF05042) are members of the EC: 1.11.2.3 class of oxidoreductases, and they also have a wide range of biological functions (Rahman et al., 2018). CLO proteins usually contain a highly conserved single calcium-binding EF hand motif, a lipid-binding domain and two invariant heme-coordinating histidine residues (Hanano et al., 2006; Kim et al., 2011; Shen et al., 2014; Rahman et al., 2018). Additionally, there is a region containing several predicted kinase sites proximal to the C-terminus (Shen et al., 2014; Song et al., 2014; Charuchinda et al., 2015), and these structures are usually important features for identifying the caleosin family and its classification. In general, there are two different *CLO* isomers in angiosperms, labeled H (high) and L (low), where H-forms contain an additional C-terminal motif of approximately 30–50 residues that is absent from L-forms, and L-caleosins evolve from H-caleosins (Khalil et al., 2014; Shen et al., 2014, 2016; Rahman et al., 2018). Among the eight CLO proteins found in *Arabidopsis*, *AtCLO1-3*, and *AtCLO8* are H-forms, and *AtCLO4-7* is an L-form (Shen et al., 2016). Caleosin is considered a structural stabilizer of lipid droplets and is named for its ability to combine with calcium (Shen et al., 2016). If caleosin has heme groups coordinated by two invariant histidine residues, it will have specific types of lipid peroxygenase activity (Hanano et al., 2006; Blée et al., 2012; Benaragama et al., 2017). Some CLO subtypes can bind to a variety of cellular bilayer membranes, such as the endoplasmic reticulum (ER) and plasmalemma, through a single transmembrane domain (Partridge and Murphy, 2009; Hanano et al., 2015; Purkrtová et al., 2015).

Soil salinity seriously affects world agricultural production (Munns and Gilliham, 2015). Salt stress is an abiotic stress factor that seriously affects the growth, development and survival of plants (Ganie et al., 2019; Xu et al., 2020). In China, saline-alkali soils account for 25% of farmland and are underutilized (Liu and Wang, 2021). Cultivating salt-tolerant plants and deeply understanding the salt tolerance mechanism of plants play an important role in agricultural production and sustainable development of the environment (Deinlein et al., 2014; Liu and Wang, 2021). Some studies have confirmed that the *CLO* gene family may be related to signal transduction and a variety of abiotic stress responses (Kim et al., 2011; Khalil et al., 2014). In *Arabidopsis*, *AtCLO1* (*ATS1*; *At4g26740*) has been found to actively participate in the degradation of storage lipids in oil bodies (OBs) (Poxleitner et al., 2006) and to have Ca^2+^-dependent peroxygenase activity, which may be related to oxylipin signaling pathways and plant defense responses (Hanano et al., 2006). *AtCLO3* (*RD20*; *At2g33380*) was significantly induced under salt, drought, and abscisic acid (ABA) stresses, and the tolerance of its mutant *rd20* to these stresses was significantly reduced (Takahashi et al., 2000; Partridge and Murphy, 2009; Aubert et al., 2010). In *Arabidopsis* overexpressing *RD20*, 13-hydroxy-9,11,15-octadecaterinoic acid (a linolenate-derived hydroxide) was enriched; the level of reactive oxygen species (ROS) increased in plants with early gibberellin-dependent flowering and ABA hypersensitivity at seed germination, indicating that *RD20* is directly related to abiotic stress (Blée et al., 2014). Compared with the wild type, *Arabidopsis* plants with high *AtCLO4* expression were less sensitive to exogenous ABA, salt and mannitol stresses, but a loss-of-function mutant (*atclo4*) was hypersensitive (Kim et al., 2011). In *OsEFA27*, the first OB calcium protein identified in rice, experimental results showed that the protein was induced by exogenous ABA (Frandsen et al., 1996). Wei et al. (2011) identified 6 *OsCLO* genes, 3 (*OsCLO-2, OsCLO-3*, and *OsCLO-6*), that can be induced by drought stress. In wheat, *CLO3* plays an important role in low-temperature stress, stomatal regulation and G (GTP-binding protein) protein signal transduction (Khalil et al., 2011).

Cotton is an important cash crop and plays an important role in the world’s textile industry (Du et al., 2018; Zhang J. et al., 2020). The yield and quality of cotton are severely impaired under exposure to various external stresses, such as salinity and drought (Zhou et al., 2014; Abdelraheem et al., 2019). The caleosin (*CLO*) gene family has been identified in *Arabidopsis* and some other species, and it has been found that it has an important relationship with signal transduction and a variety of abiotic stresses (Kim et al., 2011; Blée et al., 2014; Khalil et al., 2014). However, there are few studies on the function of *CLO* genes in cotton, and the regulatory mechanism is not clear. Therefore, it is necessary to explore the potential function of the *CLO* gene family in cotton. In this work, the members of the *CLO* gene family were identified in 15 plant species, and phylogenetic analysis was performed. The expression profiles and preliminary functions of *GhCLOs* in response to salt stress were analyzed. Virus-induced gene silencing verified that *GhCLO06* has a positive regulatory effect on salt tolerance in cotton, and this positive function may be related to the ABA signaling pathway. The results provide an important reference for further exploring the potential roles of *CLO* genes in cotton stress resistance.

## Materials and Methods

### Identification and Sequence Retrieval of *CLO* Gene Family Members

Genome and protein sequence data for *Gossypium arboreum* (CRI), *Gossypium raimondii* (JGI) and *Gossypium barbadense* (HAU) were downloaded from CottonFGD (Zhu et al., 2017), and those for *Gossypium hirsutum* (ZJU) were obtained from CottonGen (Yu et al., 2014). Eight *Arabidopsis AtCLO1-8* sequences (Shen et al., 2014) were obtained from the *Arabidopsis thaliana* TAIR website^1^. The hidden Markov model (HMM) profile (PF05042) of the conserved caleosin domain was downloaded from the Pfam database^2^. The HMMER 3.0 program (Finn et al., 2011) was used to identify all *CLO* gene family members based on the published genomes of species [*e*-value (*E*) < 10^–20^]. Searches were also performed against ten other species, namely, *Micromonas pusilla*, *Physcomitrella patens*, *Azolla filiculoides*, *Oryza sativa*, *Eucalyptus grandis*, *Glycine max*, *Populus trichocarpa*, and *Theobroma cacao* (data downloaded from phytozome_V13), *Picea abies* (data downloaded from the PlantGenIE.org website^3^), and *Ostreococcus lucimarinus* (data downloaded from the NCBI genome website^4^). Furthermore, the conserved domains of all the candidate CLO protein sequences were identified using the online Simple Modular Architecture Research Tool (SMART) (Letunic et al., 2021). The *CLO* genes (except *AtCLOs*) were named based on gene positions on the chromosomes.

Phosphorylation sites of CLO were predicted using NetPhos 3.1 (Blom et al., 1999, 2004). The isoelectric point (pI) and molecular weight (MW) of CLO proteins were analyzed by the ExPASy Proteomics Server^5^. Transmembrane domain analysis of CLO protein sequences was performed using TMHMM^6^.

### Multiple Alignment and Phylogenetic Analysis of Caleosin Proteins

To study the phylogenetic relationships among different species, multiple sequence alignments of CLO protein sequences were carried out using the Clustal X program (Larkin et al., 2007) and imaged using ESPript 3.0 (Robert and Gouet, 2014). The alignment result was employed to construct a neighbor-joining (NJ) tree by MEGA 7.0 software, 1,000 bootstrap repetitions were used to increase the reliability of interior branches, and the default values were used for other parameters (Kumar et al., 2016).

### Gene Structure and Chromosomal Distribution

To better understand the conservation of the *CLO* genes, the GSDS 2.0 program was used to analyze the structures of the *CLO* gene family (Hu et al., 2015). The gene loci of four cotton species were confirmed according to the genome annotation data and drew by TBtools software (Chen C. et al., 2020).

### Gene Duplication Events and Selection Pressure

This study used a BLASTp search (*E*-value < 1e-10) to align protein sequences in three cotton species, and the MCScanX program in TBtools was employed to perform genome collinearity analysis based on the BLASTp results (Wang et al., 2012; Chen C. et al., 2020). The circular maps of identified *CLO* gene pairs in three cotton species were displayed using TBtools software (Chen C. et al., 2020). The coding sequences of *CLO* homologous gene pairs were used to calculate the ratios of non-synonymous (*K*_*a*_) substitutions and synonymous (*K*_*s*_) substitutions by the NG methods of TBtools to evaluate the selection pressure on these gene pairs (Hurst, 2002; Chen C. et al., 2020). Normally, *K*_*a*_/*K*_*s*_ < 1 indicates purifying selection, *K*_*a*_/*K*_*s*_ = 1 indicates neutral selection, and *K*_*a*_/*K*_*s*_ > 1 indicates positive selection. The divergence times of the homologous gene pairs were estimated using the formula *t* = *K*_*s*_/2*r*, with *r* = 2.6 × 10^–9^ representing neutral substitution (Sun et al., 2019).

### Analysis of Conserved Motifs and *cis*-Acting Elements

The conserved domains of GhCLO proteins were analyzed using the online software MEME 5.1.0 with the following optimized parameters: the maximum number of motifs was set to 6, and other parameters were set to default values (Bailey et al., 2009). The *GhCLO* promoter regions containing 2,000 bp of DNA upstream of the initiation codon (ATG) were extracted from the *G. hirsutum* genome database (Yu et al., 2014). The 2,000-bp upstream regions were analyzed by PlantCARE software to detect *cis*-acting elements in the promoter regions (Lescot et al., 2002).

### Plant Materials and Treatments

The upland cotton material TM-1 was planted in greenhouse with a suitable environment (light/dark cycle: 28°C for 16 h and dark for 8 h) to explore the reaction to NaCl, PEG, methyl jasmonate (MeJA), and ABA treatment. When the seedlings reached the stage with two flat true leaves, their roots were soaked in 200 mM NaCl and 30% PEG6000, respectively. And the leaf samples were collected after 0, 1, 3, 6, 12, and 24 h of treatment. The leaves were sprayed with 100 mM MeJA and 200 mM ABA, respectively, and the leaves of three seedlings were collected from every treatment at 0, 1, 3, 6, 9, 12, and 24 h after the stress treatments. Three biological replicates were collected from each plant and immediately frozen in liquid nitrogen.

### Transcriptome Data Analysis, RNA Extraction and Quantitative RT–PCR Experiments

RNA-Seq data were obtained from the SRA database (PRJNA490626) (Hu et al., 2019). Raw RNA-seq reads were filtered using the SRAToolkit (v 2.9.2) (Leinonen et al., 2011) and trimmed by Trimmomatic (v 0.3.9) (Bolger et al., 2014) to generate clean reads, and the filtered clean RNA-seq reads were analyzed by HISAT2 (v 2.1.0) (Kim et al., 2015), SAMtools (v 1.9) (Li et al., 2009), and StringTie (v 2.0) (Pertea et al., 2015). Gene expression was measured in fragments per kilobase per million (FPKM) values, and expression levels were expressed as log_2_ (FPKM + 1) values (Chen P. et al., 2020). HemI 1.0.3.7 software was used to visualize the results (Deng et al., 2014).

A Polysaccharides and Polyphenolics-rich RNAprep Pure Plant Kit (TIANGEN, Beijing, China) was used to extract total RNA from collected samples, and the RNA samples were reverse transcribed into complementary DNA (cDNA) using the Prime Script RT Reagent Kit (TaKaRa, Japan). An ABI 7500 real-time PCR system (Applied Biosystems, United States) was used to perform qRT–PCR (Promega, Madison, WI, United States) with three biological replicates. The qRT–PCR primers of *GhCLOs* were listed in Supplementary Table 1, and *GhACTIN* was used as a constituent expression control in qRT–PCR experiments. The results were calculated using the 2^–Δ^ ^Δ^ *^Ct^* relative quantitative method (Livak and Schmittgen, 2001).

### Virus-Induced Gene Silencing of the *GhCLO06* Gene in Cotton

Virus-induced gene silencing (VIGS) assays were carried out by using *tobacco rattle virus* (TRV) vectors (Burch-Smith et al., 2004). The TRV system contains two vectors, pTRV1 (pYL192) and pTRV2 (pYL156), and the gene was silenced by inserting the target gene fragment of the pTRV2 vector. The web-based SGN VIGS Tool^7^ was used to design the silenced fragment of *GhCLO06*. The fragment was PCR-amplified and cloned into the pTRV2 vector to produce pTRV2:*GhCLO06* constructs. Oligo 7 software was used to design primers (Supplementary Table 1) (Rychlik, 2007). The pTRV2:00, pTRV2:*GhCLO06*, pTRV2:*GhPDS*, and pTRV1 (pYL192) constructs were transformed into *Agrobacterium tumefaciens* strain LBA4404. The above cultures were collected by centrifugation and resuspended in infiltration buffer (10 mM MgCl2, 10 mM MES, and 200 μM acetosyringone) to a 1.5 OD600 value. After incubation at room temperature for 3 h, the first three kinds of *Agrobacterium* suspensions containing vectors of pTRV2:00, pTRV2:*GhCLO06*, and pTRV2:*GhPDS* were mixed with the same amount of *Agrobacterium* suspension holding the vector of pTRV1 (pYL192). Cotton seedlings were grown at 16 h/8 h (light/dark) at 25°C. After the cotyledons of cotton were flattened, the seedlings were infiltrated with mixed culture using a 1 ml syringe. Leaves were collected for RNA extraction and interference efficiency detection. At the three-leaf stage, silenced plant roots were soaked in 200 mM NaCl solution, and deionized water was used as a control. The treatments were repeated three times. Cotton plant wilting rates were calculated as the percentage of wilted plants to the total stressed plants. Malondialdehyde (MDA), superoxide dismutase (SOD), and peroxidase (POD) were extracted and identified according to standard methods (Solarbio, Beijing, China). The error bars represent the standard deviations of three biological replicates.

## Results

### Identification of *CLO* Genes in Green Plants

**FIGURE 1:**
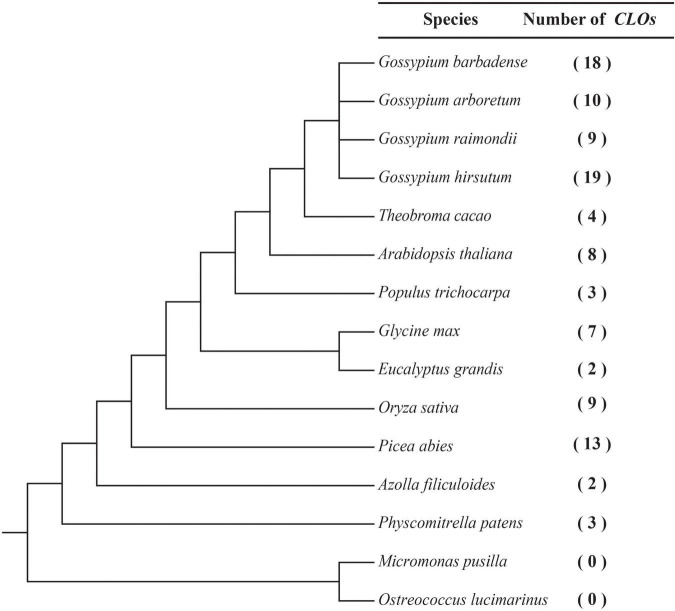
Inferred phylogenetic relationships among 15 species. The number of *CLO* genes detected in each genome is indicated on the right.

To identify the *CLO* genes in green plants, 107 *CLO* genes were identified in *G. hirsutum*, *G. barbadense*, *G. raimondii*, *GossypiumArboretum*, and 11 other species (Figure 1 and Supplementary Table 2) including green algae (*O. lucimarinus*, *M. pusilla*), a bryophyte (*P. patens*), a pteridophyte (*A. filiculoides*), a gymnosperm (*P. abies*), a monocot (*O. sativa*), and eudicots (*E. grandis*, *G. max*, *P. trichocarpa*, *A. thaliana*, and *T. cacao*). The *CLO* genes identified in these species were named with a species-specific letter as a prefix and a numerical suffix, which was based on the chromosomal position of the gene (Supplementary Table 2). The evolutionary relationships of these 15 species and the number of corresponding *CLO* genes were determined (Figure 1). No *CLO* genes were identified in the green algae (*O. lucimarinus* and *M. pusilla*). Among the six plant evolutionary lineages from lower plants to higher plants, the size of the *CLO* gene family varied from 0 to 19 members (Supplementary Table 2). Three species had more than 10 members, five species had 7–10 members, and the other species had fewer than 5 members.

In total, 19, 18, 9, and 10 *CLO* genes were identified in *G. hirsutum*, *G. barbadense*, *G. raimondii*, and *G. arboreum*, respectively. The protein lengths of GhCLO, GbCLO, GaCLO, and GrCLO members varied from 209 to 236 (aa), 143 to 296 (aa), 202 to 285 (aa), and 202 to 287 (aa), respectively. The physicochemical properties of *CLOs* showed that the pI of the protein was between 5.812 and 9.268, and the molecular weight was between 16.16 and 33.369 kDa. Subcellular localization results showed that CLO proteins were mainly located in the cytoplasm and periplasmic region (Supplementary Table 3).

### Phylogenetic Analysis of the *CLO* Gene Family

To study the evolutionary relationships of the *CLO* gene family, referring to a study in *Arabidopsis* (Shen et al., 2014), 107 CLO proteins were classified into three categories (named class I, class II, and class III) (Supplementary Figure 1). Among the 3 types of CLO proteins, class I was clustered with H (high)-caleosins of *Arabidopsis*, which was also the largest category, with 66 CLO proteins (11 species). Class II contained 37 proteins (11 species), which were clustered with L (low)-caleosins of *A. thaliana*. In addition to these 2 categories, a unique class, class III, was formed, which contained 4 *CLO* genes, including all bryophyte (*P. patents*) *CLO* genes (3) and one pteridophyte (*A. filiculoides*) *CLO* gene (Supplementary Figure 1). Except for those in gymnosperms (*P. abies*), the *CLO* genes were distributed in the 2 clusters (class I and class II). These results showed that in the long-term evolutionary process, the *CLO* gene family formed certain species differences from lower plants to higher plants, but it remained highly conserved within species.

### Exon–Intron Structure, Conserved Motif Analysis and Multiple Sequence Alignments of *CLO* Genes in Cotton

To further investigate the phylogenetic relationships and understand the structural diversity and structural characteristics of *CLO* genes, the intron/exon structures of each *CLO* from *G. hirsutum, G. barbadense, G. arboreum*, and *G. raimondii* were analyzed (Supplementary Figure 2). The numbers of exons and introns in *CLO* genes in cotton ranged from 5 to 9 and 4 to 8, respectively. Eighty percent (45/56) of the *CLO* genes contained 6 exons and 5 introns, except for *GaCLO04* and *GbCLO16* (5 exons and 4 introns); *GrCLO01*, *GrCLO05*, *GaCLO08*, *GaCLO10*, *GhCLO05*, *GbCLO01*, *GbCLO05*, and *GbCLO10* (6 exons and 5 introns); and *GbCLO01* (9 exons and 8 introns). The number of exons/introns is related to the organism’s ability to adapt to adverse environmental conditions, structural divergence and functional differentiation (Xu et al., 2012; Shang et al., 2017). Motif analysis of 56 CLO amino acid sequences of 4 cotton genomes (*G. hirsutum, G. barbadense, G. arboreum*, and *G. raimondii*) was carried out by the MEME program. Six motifs (motifs 1–6) were identified in the CLO proteins (Supplementary Figure 2): 68% (38/56) contained motif 4; class I contained motif 1, motif 2 and motif 6; and class II contained motif 4, motif 1, motif 6 and motif 3 but not the GbCLO15. These results showed that the *CLO* gene family was highly conserved in terms of protein sequence and gene structure, but the structural differences of some genes might also lead to functional differentiation.

**FIGURE 2:**
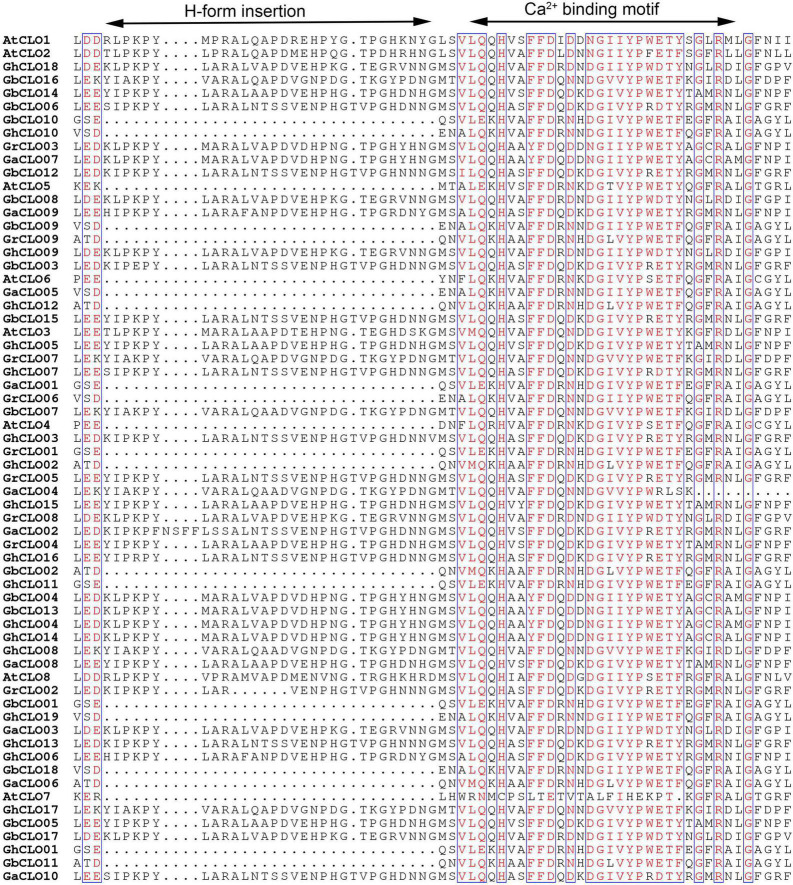
Multiple alignments of CLO proteins in cotton.

Multiple alignments were performed using 56 caleosin protein sequences from 4 cotton species and 8 caleosin protein sequences from *A. thaliana*. The alignments of H-form insertions and EF-hand Ca^2+^-binding motifs are shown in Figure 2. Among the 64 CLO proteins, 42 contained H-form insertions and were called H-caleosins, and the others were called L-caleosins (Supplementary Table 3). The N-terminus was the main difference between L- and H-isoform caleosins. An insertion in the N-terminus of the middle hydrophobic region of the H-isoform made its N-terminus larger (Naested et al., 2000; Hanano et al., 2006; Shen et al., 2014). The EF-hand Ca^2+^-binding motifs of GaCLO04 were partially lost, resulting in the incompleteness of the domain, which might cause it to lose its ability to bind calcium (Shen et al., 2016).

### Chromosomal Distribution, Gene Duplication and Selection Pressure

The chromosomal distributions of *GrCLO*, *GaCLO*, *GbCLO*, and *GhCLO* genes were visualized according to the genomic positions of 56 cotton *CLO* genes (Supplementary Figure 3). Ten *GaCLO* genes were distributed on scaffolds A02, A09, A10, A12 and 2, and 9 *GrCLO* genes of *G. raimondii* were distributed on chromosomes D02, D06, D08, D11, and D12. Among the 19 *CLO* genes in *G. hirsutum*, 10 came from the At subgenome and 9 from the Dt subgenome. Nine *CLO* genes were identified in the At and Dt genomes of *G. barbadense.* The number (18) of *CLOs* identified in allotetraploid *G. barbadense* was one less than the sum of the numbers in the two diploid cotton species (*G. raimondii* and *G. arboreum*). The distributions of *CLO* genes in tetraploid *G. hirsutum* and *G. barbadense* were similar, but there were differences in chromosome distribution corresponding to the diploid species (*G. arboreum* and *G. raimondii*).

**FIGURE 3:**
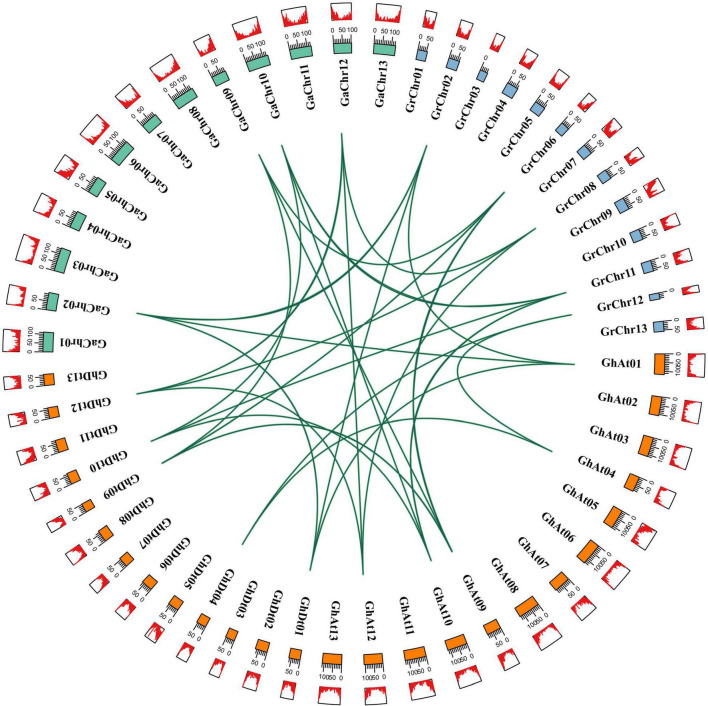
*CLO* homologous gene pairs among *Gossypium arboreum*, *Gossypium raimondii*, and *Gossypium hirsutum*. Orange, blue and green represent chromosomes of *G. hirsutum*, *G. raimondii* and *G. arboreum*, respectively; red represents the density of genes on chromosomes.

The allotetraploid cotton species *G. hirsutum* is derived from the hybridization of two diploid cotton species (*G. arboreum* and *G. raimondii*) (Zhang Q. et al., 2020). The types of gene replication mainly include tandem duplication, segmental duplication and whole-genome duplication (WGD) (Cannon et al., 2004). BLASTp and MCScanX software were used for homologous sequence alignment and collinearity analysis of *CLO* genes in four cotton species, and the results were used to identify the duplication types of tetraploid cotton species (*G. hirsutum* and *G. barbadense*) (Wang et al., 2012). The analysis of the gene duplication types of *G. hirsutum* and *G. barbadense* showed that *CLO* family genes mainly came from segmental duplication or WGD (Supplementary Table 4), which indicated that segmental duplication or WGD played an important role in the evolution of the *CLO* gene family. Homologous gene pairs were determined from the results of the gene sequence comparison program BLASTp, the genomes of *G. hirsutum, G. arboreum*, and *G. raimondii* were analyzed for collinearity, and the results were visualized (Figure 3). Most of the non-synonymous (*K*_*a*_)/synonymous (*K*_*s*_) values of all identified *CLO* homologous gene pairs were less than 1, and only 3 pairs [(*GhCLO*05/*GaCLO08*), (*GhCLO10/GaCLO05*), and (*GhCLO10/GhCLO19*)] showed values greater than 1, indicating that these 3 gene pairs might undergo positive selection, resulting in gene differentiation and new biological functions, while other genes were under strong purifying selection (Supplementary Table 5). The divergence time of *CLO* genes in *G. raimondii, G. arboreum* and the two subgenomes of *G. hirsutum* was predicted by the formula “*t* = *K*_*s*_/2*r*” (*r* = 2.6 × 10^–9^) (Zhang et al., 2015). The results showed that except for the two gene pairs [(*GhCLO05/GaCLO08*) and (*GhCLO07/GaCLO10*)], the divergence time of *CLOs* of three cotton species might have occurred 1.203 to 50.848 million years ago (MYA) (Supplementary Table 5).

### Analysis of *cis*-Elements in Predicted Promoter Regions of *GhCLOs*

**FIGURE 4:**
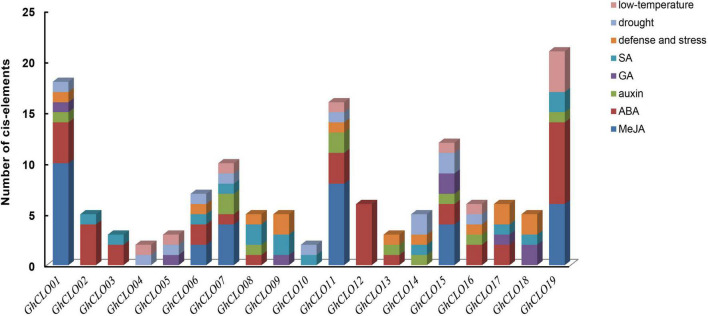
*Cis*-elements of *GhCLO* genes in promoter regions. The numbers of different *cis*-elements are presented as bars, with similar *cis*-elements shown by the same colors.

To better study the possible functions of *GhCLOs* in abiotic stress and hormone regulation, the 2,000-bp promoter regions of 19 *GhCLO* genes were analyzed by PlantCARE (Figure 4 and Supplementary Table 6). Among the 5 hormones, the number of *cis*-elements related to ABA hormone was the largest (38), distributed in 13 *CLO* promoters, and followed by MeJA, including 34 *cis*-elements. In addition, there were 3 stress-related elements, namely, defense and stress (TC-rich repeats), drought (MBS) and low temperature (LTR), with numbers of 13, 12, and 10, respectively. Low-temperature stress elements were distributed in the promoters of 8 genes, and the other 2 types of elements were distributed in the promoters of 10 *CLO* genes. In addition, there were 16 elements (W-box) in the *GhCLO* family and distributed in the promoters of 10 *GhCLO* genes (Supplementary Table 6). Studies have shown that the W-box plays an important role in the response to salt stress (Xu et al., 2018; Yao et al., 2020). These results revealed that *GhCLOs* might be related to hormones and multiple abiotic stresses.

### Expression Profiles of *GhCLO* Genes in Different Tissues and Under Different Abiotic Stresses

**FIGURE 5:**
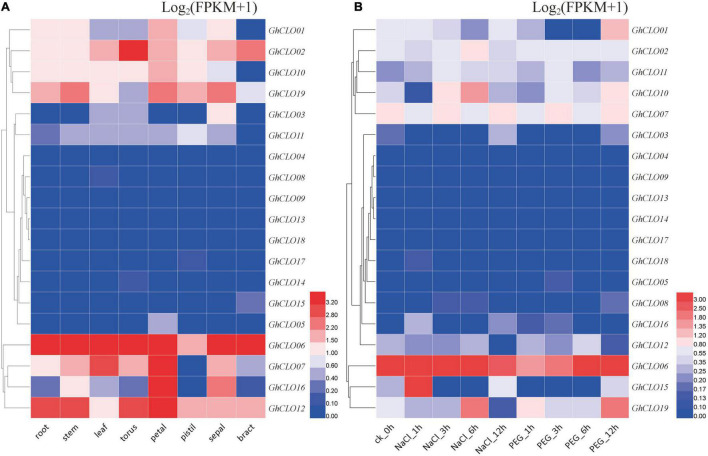
Expression profiles of *GhCLOs* in different tissues **(A)** and responses to different stresses **(B)**. The tissues or treatments are shown at the bottom, the genes are shown on the right, and the phylogenetic relationships are shown on the left. The color scale in the upper right corner of the heatmap represents the FPKM values, which were standardized by log_2_(FPKM + 1). FPKM, fragments per kilobase per million.

To deeply study the potential biological functions of the *GhCLO* gene family, their tissue specificity in cotton was analyzed, including root, steam, leaf, torus, petal, pistil, sepal, and bract tissues (Figure 5A and Supplementary Table 7). *GhCLO06* was highly expressed in many tissues; *GhCLO02*, *GhCLO07*, *GhCLO16*, and *GhCLO12* were highly expressed in individual tissues, but the expressions of 9 *GhCLO* genes, including *GhCLO04*, *GhCLO05*, *GhCLO08*, *GhCLO09*, *GhCLO13*, *GhCLO14*, *GhCLO18*, *GhCLO17*, and *GhCLO15*, were very low in the eight tissues. The differences of tissue-specific expression indicated that the functions of the *GhCLOs* might have been differentiated in the long-term evolutionary process, and its specific biological functions might diverge among tissues.

Transcriptome data were also used to analyze the *GhCLO* gene family under salt and drought stresses (Figure 5B and Supplementary Table 7). There were significant differences in the expression trends of *GhCLO* genes after salt and drought treatments. *GhCLO01*, *GhCLO02*, *GhCLO07*, *GhCLO10*, and *GhCLO11* showed varying trends after treatment, and the expression levels were relatively low in different treatment periods. The expression levels of *GhCLO03*, *GhCLO04*, *GhCLO05*, *GhCLO08*, *GhCLO09*, *GhCLO13*, *GhCLO14*, *GhCLO16*, *GhCLO17*, and *GhCLO18* were very low at different stages after treatment. *GhCLO06* was induced after salt and drought treatments, and its expression was higher at different stages after treatment. These results suggested that *GhCLOs* might have functional differences in response to abiotic stress.

### Quantitative RT–PCR Experiments of *GhCLO* Genes Under Salt, Drought, Abscisic Acid, and Methyl Jasmonate Treatments

**FIGURE 6:**
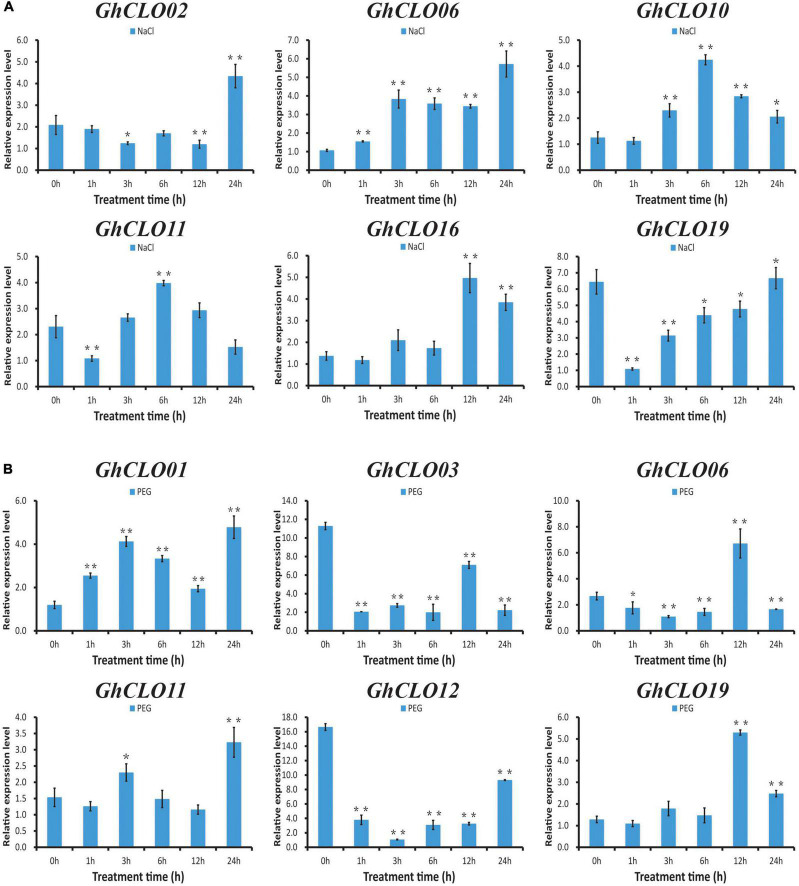
Relative expression levels of *GhCLO* genes under salt and PEG treatments detected by qRT-PCR. **(A)** The relative expression levels of selected genes under control conditions (water) and salt treatment. **(B)** The relative expression levels of selected genes under control conditions (water) and PEG treatment. Error bars show the standard deviations of three biological replicates. *P < 0.05, **P < 0.01 (*t*-test).

Based on the *cis*-elements in the promoter of the *GhCLO* genes and the results of previous studies, 6 *GhCLO* genes were selected for qRT–PCR experiments under salt treatment (Figure 6A). The expressions of *GhCLO10* and *GhCLO11* first increased and then decreased after treatment, and the expression levels were the highest at 6 h. *GhCLO02* and *GhCLO16* were significantly induced at 24 and 12 h after treatment, respectively. The expression of *GhCLO06* was higher than that of the control (0 h) in different treatment periods. *GhCLO09*, *GhCLO13*, *GhCLO14*, *GhCLO16*, *GhCLO17*, and *GhCLO18* were selected for drought treatment analysis (Figure 6B). The expression levels of *GhCLO01* and *GhCLO11* were higher at 3 and 24 h after treatment, respectively. The expression levels of *GhCLO03* and *GhCLO12* in different periods after drought stress treatment were lower than those in the control (0 h). *GhCLO06* and *GhCLO19* were induced at 12 h after treatment. These results showed that *GhCLO* genes had different expression patterns in response to salt and drought stresses.

**FIGURE 7:**
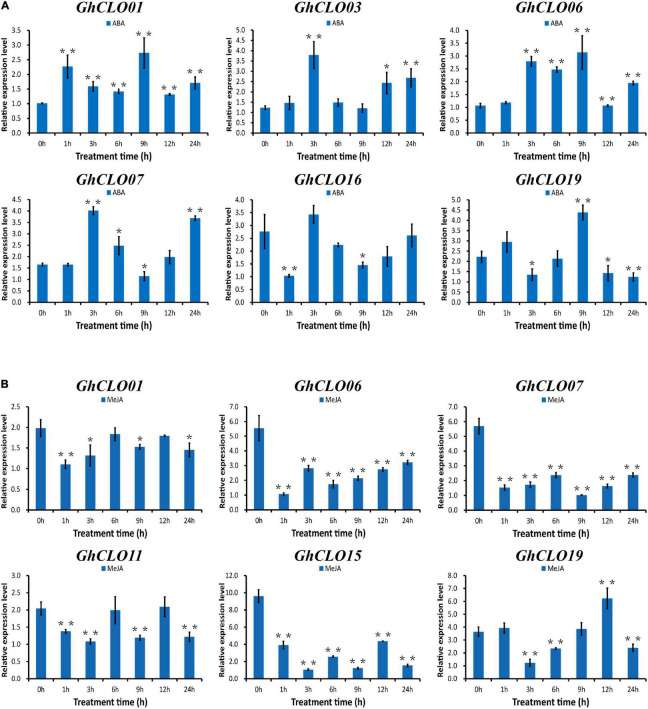
Relative expression levels of *GhCLO* genes under ABA and MeJA treatments. Two true leaves were sprayed with 200 mM ABA and 100 mM MeJA, and water was used as a blank control. **(A)** The relative expression levels of selected genes under control conditions (water) and ABA treatment. **(B)** The relative expression levels of selected genes under control conditions (water) and MeJA treatment. Error bars show the standard deviations of three biological replicates. *P < 0.05, **P < 0.01 (*t*-test).

In the prediction of *cis*-elements in the promoter, it was found that the numbers of *cis*-elements related to ABA and MeJA hormones were larger (Supplementary Table 6). Studies have shown that ABA and MeJA in plants play critical roles in the response to a variety of abiotic stresses, such as high salinity, drought stress and cold (Reyes and Chua, 2007; Su et al., 2011; Chen et al., 2019; Tavallali and Karimi, 2019). Six *GhCLO* genes were selected for exogenous ABA and MeJA stress analysis (Figure 7). After exogenous ABA treatment, the expression levels of *GhCLO01*, *GhCLO06*, and *GhCLO19* were the highest at 9 h. The expression levels of *GhCLO03*, *GhCLO07*, and *GhCLO16* peaked at 3 h (Figure 7A). After treatment with exogenous MeJA, the expression levels of *GhCLO06*, *GhCLO07*, and *GhCLO15* were significantly lower than those in the control (0 h). The relative expression level of *GhCLO19* increased gradually 3–12 h after treatment (Figure 7B). These results suggested that *GhCLOs* might be regulated by MeJA or ABA.

### Silencing of *GhCLO06* in Cotton Hinders Cotton Resistance to Salt Stress

By combining transcriptome data and qRT–PCR analysis, it was found that the *GhCLO06* was responded positively to salt and drought stresses (Figures 5B, 6A). Previous studies have shown that a *CLO* gene *AtCLO3* (*RD20*) is closely related to salt stress and ABA (Takahashi et al., 2000; Aubert et al., 2010; Blée et al., 2014). These results suggested that *GhCLO06* might play an important role in response to salt stress.

**FIGURE 8:**
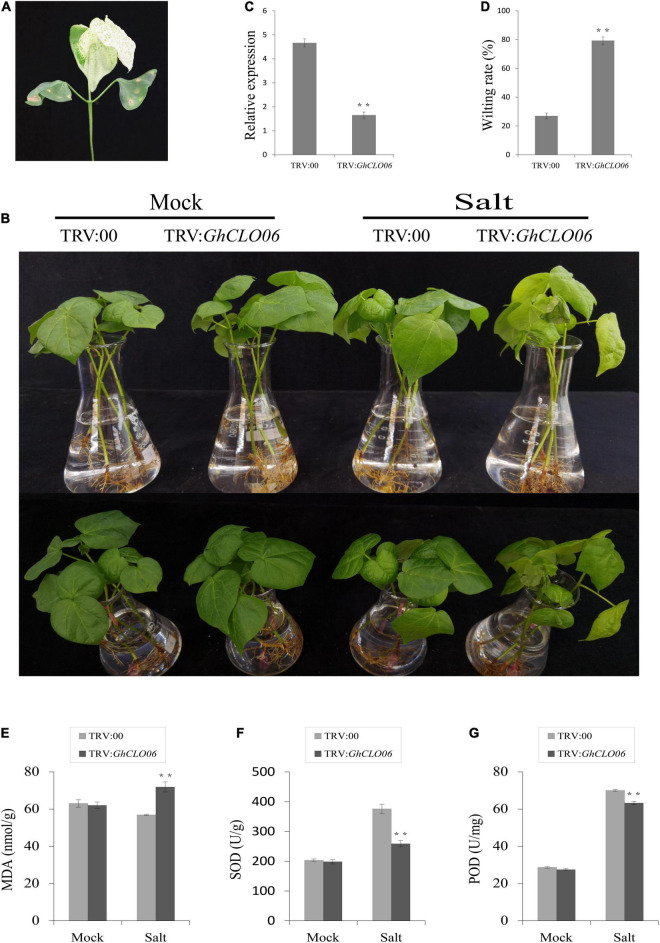
**Silencing of *GhCLO06*** decreased resistance to salt stress in cotton. **(A)** Leaf whitening of TRV:*GhPDS* (positive control). **(B)** Phenotype of the control plants (TRV:00) and gene-silenced plants with *GhCLO06* gene silencing (TRV:*GhCLO06*) under normal conditions (Mock) and salt stress for 4 days. **(C)** Relative expression of *GhCLO06* in the control plants (TRV:00) and gene-silenced plants (TRV:*GhCLO06*) determined via qPCR analysis. **(D–G)** Physiological parameters were quantified in plants cultivated under normal (mock) and salt stress conditions. **(D)** Wilting rate; **(E)** malondialdehyde (MDA) content; **(F)** superoxide dismutase (SOD) activity; **(G)** peroxidase (POD) activity. Error bars indicate standard deviations estimated by three independent experiments. **P < 0.01 (*t*-test).

**FIGURE 9:**
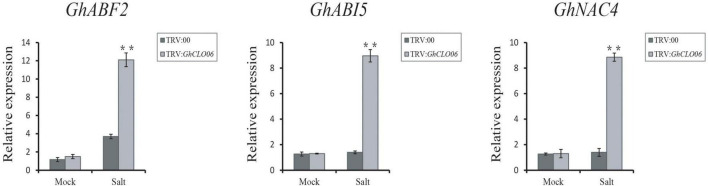
Abscisic acid-responsive genes *GhABF2*, *GhABI5*, and *GhNAC4* in control plants and TRV:*GhCLO06* plants in normal (mock) and salt environments; ABA, abscisic acid. Error bars indicate standard deviations estimated by three independent experiments. **P < 0.01 (*t*-test).

To further verify our prediction, we carried out a VIGS experiment to verify the role of the *GhCLO06* gene in cotton under salt stress. Ten days after infection, plants in which the *GhPDS* gene was silenced (positive control) exhibited the albino phenotype (Figure 8A), which indicated the effectiveness of the experiment. qRT–PCR showed that the relative expression level of TRV:*GhCLO06* decreased significantly compared with that of the control plant TRV:00, indicating that the gene was significantly inhibited (Figures 8B,C). To estimate the salt resistance of the target gene-silenced cotton plants, TRV:00 and TRV:*GhCLO06* plants were treated with 200 mM NaCl and deionized water (control) for 4 days. Compared with the control plants (TRV: 00), the leaves of TRV:*GhCLO06* cotton plants under salt treatment displayed considerable damage, including yellowing and wilting (Figure 8B). The wilting rate of TRV:*GhCLO06*-silenced plants was significantly higher than that of control plants (TRV:00) (Figure 8D). In addition, physiological indexes such as MDA content and SOD and POD activity in the leaves of gene-silenced (TRV:*GhCLO06*) plants and control (TRV:00) plants were investigated (Figures 8E–G). No significant differences in the MDA content or SOD and POD activities were observed between control (TRV:00) plants and target gene-silenced (TRV:*GhCLO06*) plants under normal conditions. Under salt stress for 4 days, compared with the control (TRV:00) plants, the MDA content of the target gene-silenced (TRV:*GhCLO06*) plants increased significantly, but the activities of SOD and POD decreased significantly. Under salt stress, the expressions of the *GhABF2, GhABI5*, and *GhNAC4* genes related to ABA signal regulation were significantly upregulated, and the transcriptions of three genes in the target gene-silenced (TRV:*GhCLO06*) plants were 6 times higher than that before treatment (Figure 9).

## Discussion

### Comparative Genomic Analysis of the *CLO* Gene Family in Green Plants

The completion of whole-genome sequencing provides support for the whole-genome identification and evolutionary analysis of gene families in many plants. In this study, the *CLO* families of 13 terrestrial plants and 2 green algal species were analyzed, and a total of 107 *CLO* genes were identified. Interestingly, *CLO* genes were detected in terrestrial plants but not in the two tested green algal species (Figure 1 and Supplementary Table 2). We speculated that the current *CLO* gene family may have evolved mainly in terrestrial plants. During the evolution of terrestrial plants, the size of the *CLO* gene family varied from 2 to 19 members. Three species had more than 10 members, five species had 7–10 members, and the other species had fewer than 5 members (Figure 1). Studies have found that the *CLO* gene family is widespread in terrestrial organisms and plays a role in a variety of stress responses (Wei et al., 2011; Hanano et al., 2015; Purkrtová et al., 2015). The size of the gene family in species evolution might be closely related to its species-specific function. These results indicated that the *CLO* gene family may have experienced specific biological functional differentiation during evolution in terrestrial organisms, and species-specific expansion has occurred after the evolution of these species. This phenomenon also occurred in other gene families, such as the *HMGS* gene family (Liu et al., 2019).

### Phylogeny, Gene Structure, and Expansion of *CLO* Genes in Cotton

A total of 19, 18, 10, and 9 *CLO* genes were identified in *G. hirsutum*, *G. barbadense*, *G. arboreum*, and *G. raimondii*, respectively (Supplementary Table 2). Allotetraploid cotton is the result of genomic hybridization and doubling approximately 1–1.5 MYA (Wendel and Cronn, 2003; Li et al., 2015), and gene loss is most likely an ongoing process in allotetraploid cotton (Zhang et al., 2015), which may have resulted in *G. barbadense* (18) lacking a *CLO* gene compared with *G. hirsutum* (19) and provides strong support for the study of cotton polyploidy.

To investigate the conservation of the *CLO* gene sequences in cotton, exon–intron structures and conserved motifs were analyzed (Supplementary Table 3). The numbers of exons and introns of *CLO* genes in cotton ranged from 5 to 9 and from 4 to 8, respectively. Eighty percent (45/56) of CLO proteins contained 6 exons and 5 introns. In addition, among the six types of motifs identified, the motifs on the same branch in the four cotton species showed a high degree of conservation (Supplementary Figure 2). The structural similarity of the *CLO* gene family reveals structural conservation in evolution, and the structural differences among individual genes also reflect diversity in evolution.

Polyploidy is a manifestation of plant adaptability to the environment, and it is also an important mechanism of new species formation (Ramsey and Schemske, 1998). To further study the evolutionary relationship between two diploid species (*G. arboreum* and *G. raimondii*) and allotetraploid species (*G. hirsutum* and *G. barbadense*), we analyzed the chromosomal distribution of *CLO* genes and gene duplication events (Supplementary Figure 3 and Supplementary Table 4). The results showed that the chromosomal distributions of *CLO* genes in *G. arboreum* and the corresponding At subgenome of allotetraploid cotton were not identical, which may be caused by chromosome translocation in the process of tetraploid cotton speciation (Hu et al., 2019). The chromosomal locations of *CLO* genes in *G. raimondii* and the corresponding Dt subgenome of allotetraploid cotton were highly consistent (Supplementary Figure 3), illustrating that *CLO* genes in the allotetraploid cotton Dt subgenome were highly conserved in the process of evolution. A similar chromosome evolutionary distribution was found in the *RPD3* gene family (Zhang J. et al., 2020). The replication of a single gene, chromosome or genome is the main force of plant genome evolution (Paterson et al., 2012). The duplication types of 37 genes in *G. hirsutum* and *G. barbadense* showed that 35 *CLO* genes were formed by WGD or segmental duplication based on collinearity analysis. The cotton *NF-YA*, *NHX*, and *GT47* families showed enlargement as a result of WGD and segmental duplication (Wu et al., 2019; Fu et al., 2020; Zhang Q. et al., 2020). Studies have reported that cotton has a decaploid ancestor, which has experienced an extremely complex polyploid process (Wang et al., 2016). A- and D-genome diploid cotton began to differentiate from a common ancestor 5–10 MYA (Hu et al., 2019). Subsequently, *G. hirsutum* evolved from the hybridization of two diploid cotton plants approximately 1–2 MYA (Zhang et al., 2015). In *G. hirsutum*, the deduced divergence times of most *CLO* homologous gene pairs are between 4.37 and 14.56 MYA (Supplementary Table 5), accompanied by the differentiation of ancestral genomes of A and D, which is similar to the pattern observed for the *RPD3* family (Zhang J. et al., 2020). The predicted divergence time of some genes ranged from 16.1 to 50.8 MYA, and the divergence of these genes might have gone through the diploid ancestor period of cotton or even occurred in the early decaploid ancestor period of cotton. In addition, through analysis of selective stress in the evolution of the *CLO* gene family, the results showed that the *K*_*a*_/*K*_*s*_ ratios of almost all gene pairs were less than 1 (Supplementary Table 5), indicating that the *CLO* gene family was under strong purifying selection during long-term evolution and is functionally conserved.

### Functional Analysis of *GhCLO06* in Upland Cotton

*CLO* family genes are closely related to abiotic stress and signal transduction (Khalil et al., 2011; Kim et al., 2011). After salt treatment, the expression trend of *GhCLO10* and *GhCLO11* first increased and then decreased, *GhCLO06* was induced, and the expression level in each period was higher than that in the control (water). After drought treatment, compared with those in the control, the expression levels of *GhCLO03* and *GhCLO12* decreased, and *GhCLO06* and *GhCLO19* were significantly induced at 12 h (Figure 6). The above results suggest that these *GhCLO* genes respond to salt and drought stress, and the difference in gene expression levels may be related to the importance of participating in the stress response.

In eukaryotes, transcriptional regulation is the main mechanism of gene expression regulation, and *cis*-acting elements are involved in the transcriptional regulation of genes (Ding et al., 2018; Chen P. et al., 2020; Yang et al., 2020). In general, gene expression depends on the presence or absence of these elements (Priest et al., 2009). In the prediction of *cis*-elements in the promoter, it was found that the numbers of *cis*-elements related to ABA and MeJA were the larger (Figure 4 and Supplementary Table 6). MeJA and ABA not only regulate plant growth and development but also participate in plant defense responses to environmental stress, such as mechanical injury and osmotic stress (Ellis and Turner, 2001; Anderson et al., 2004). After ABA stress treatment, the six genes were induced to varying degrees; after MeJA stress, the expression levels of *GhCLO06*, *GhCLO07*, and *GhCLO15* were significantly lower than those of the control (0 h) (Figure 7). The differences in the expression levels of *GhCLO* genes revealed that *GhCLO* genes may adopt different response patterns under the stimulation of exogenous ABA and MeJA. In addition, through the analysis of transcriptome data under salt and PEG stress, it was found that the relative expression level of *GhCLO06* in each period was significantly higher than that of other genes (Figure 5B), indicating that *GhCLO06* may play a more important role in salt and drought stress responses than other *GhCLO* genes.

VIGS-TRV is an important technology for studying the gene function of cotton. The TRV vector is widely used in the study of functional genes related to the abiotic stress response of cotton (Cai et al., 2019; Zhang Q. et al., 2020). After salt stress, the target gene-silenced plants (TRV:*GhCLO06*) exhibited obvious yellowing and wilting (Figure 8B). Malondialdehyde (MDA), superoxide dismutase (SOD), and peroxidase (POD) are important indicators of cell oxidative damage (Mittler, 2006; Li et al., 2020). A large number of studies have shown that salt stress and other factors can lead to the accumulation of reactive oxygen species (ROS), and superoxide dismutase play an important role in the clearance of ROS (Miller et al., 2008, 2010). After salt stress, the MDA content, SOD and POD activities of gene silenced plants (TRV:*GhCLO06*) increased. Compared with that in the control plants (TRV:00), the MDA content in gene silenced plants (TRV:*GhCLO06*) showed higher accumulation, but the activities of SOD and POD decreased significantly in gene silenced plants (TRV:*GhCLO06*) (Figures 8E–G). Taken together, these findings demonstrated that *GhCLO06* was a positive regulator of salt tolerance in plant.

In addition to ROS, gene tolerance to salt stress may also involve other physiological and biochemical mechanisms (Yu et al., 2020), among which ABA related to salt stress has been widely studied (Jia et al., 2002; Perin et al., 2019; Zhang Q. et al., 2020). In *Arabidopsis*, *AtCLO3* and *AtCLO4* have been confirmed to be related to ABA signal transduction (Kim et al., 2011; Blée et al., 2014). *ABF2* (Liang et al., 2016), *ABI5* (Skubacz et al., 2016), and *NAC4* (Trishla and Kirti, 2021) are considered to be important genes for ABA signal regulation. After salt stress, the expression levels of *GhABF2*, *GhABI5*, and *GhNAC4* in gene silenced plants were significantly upregulated (Figure 9). These results suggested that *GhCLO06* might regulate salt tolerance by activating the ABA signaling pathway after salt stress.

## Conclusion

In this work, a total of 107 *CLO* genes were obtained from the whole-genome identification of 15 plant species. *CLO* genes are ubiquitous in terrestrial plants but may be lacking in some green algal species. In addition, the gene structure, phylogeny and biological characteristics of *CLO* family members of four cotton species were systematically analyzed. qRT–PCR analysis suggested that some *CLO* genes might play important roles in the cotton response to salt stress. This research also revealed that the *GhCLO06* gene might play a positive role of salt tolerance and might be regulated by ABA signaling pathway in cotton. Further studies on the role of ABA homeostasis under salt stress will help clarify the comprehensive effect of the cotton *CLO* gene on salt tolerance.

## Data Availability Statement

The original contributions presented in the study are included in the article/Supplementary Material, further inquiries can be directed to the corresponding authors.

## Author Contributions

XF and HTW conceived and designed the study and prepared the manuscript. XF, YY, MK, HLW, and BL performed the experiments. BW, LM, JL, and PH assisted with the analysis and interpretation of the data. SY participated in the design of the experiments and provided a critical review. All authors have read, edited, and approved the current version of the manuscript.

## Conflict of Interest

The authors declare that the research was conducted in the absence of any commercial or financial relationships that could be construed as a potential conflict of interest.

## Publisher’s Note

All claims expressed in this article are solely those of the authors and do not necessarily represent those of their affiliated organizations, or those of the publisher, the editors and the reviewers. Any product that may be evaluated in this article, or claim that may be made by its manufacturer, is not guaranteed or endorsed by the publisher.

## Footnotes

1
https://www.Arabidopsis.org/

2
http://pfam.xfam.org/

3
https://beta.plantgenie.org/

4
https://www.ncbi.nlm.nih.gov/genome/

5
https://web.expasy.org/protscale/

6
http://www.cbs.dtu.dk/services/TMHMM/

7
https://vigs.solgenomics.net/

## Supplementary Material

Supplementary Figure 1 Phylogenetic tree of the CLO proteins in 13 species. Three categories of CLO proteins are indicated using different colors by iTOL.

Supplementary Figure 2 Phylogenetically aligned conserved motif and gene structure analysis of *CLO* genes in four cotton species.

Supplementary Figure 3 Distribution of *CLOs* among the chromosomes of four cotton species. Panels **(A–D)** represent the chromosomal locations of *CLO* genes from *Gossypium hirsutum* **(A)**, *Gossypium barbadense* **(B)**, *Gossypium arboreum* **(C)**, and *Gossypium raimondii* **(D)**, respectively. The chromosome numbers are shown on the left side of each vertical bar.

Supplementary Table 1 A list of the primers used in this study.

Supplementary Table 2 Nomenclature and sequencing of *CLO* genes.

Supplementary Table 3 Detailed physicochemical characteristics of CLO proteins in *G. hirsutum*, *G. barbadense*, *G. arboreum*, and *G. raimondii*.

Supplementary Table 4 Duplicate type of *CLOs* in *G. hirsutum* and *G. barbadense*.

Supplementary Table 5 *K*_*a*_/*K*_*s*_ ratios and occurrence times of segmentally duplicated *CLO* gene pairs in three cotton species.

Supplementary Table 6 Statistical results of phytohormones-responsive *cis*-acting elements in the promoter segments of *CLOs*.

Supplementary Table 7 The FPKM value of *GhCLO* genes in different tissues and under two different abiotic stresses.
