# Supplementary figures and images for: Evolution and Stress Responses of CLO Genes and Potential Function of the GhCLO06 Gene in Salt Resistance of Cotton
Source: Front Plant Sci. 2022 Jan 17;12:801239. doi: 10.3389/fpls.2021.801239 (PMC8802827; doi:10.3389/fpls.2021.801239)

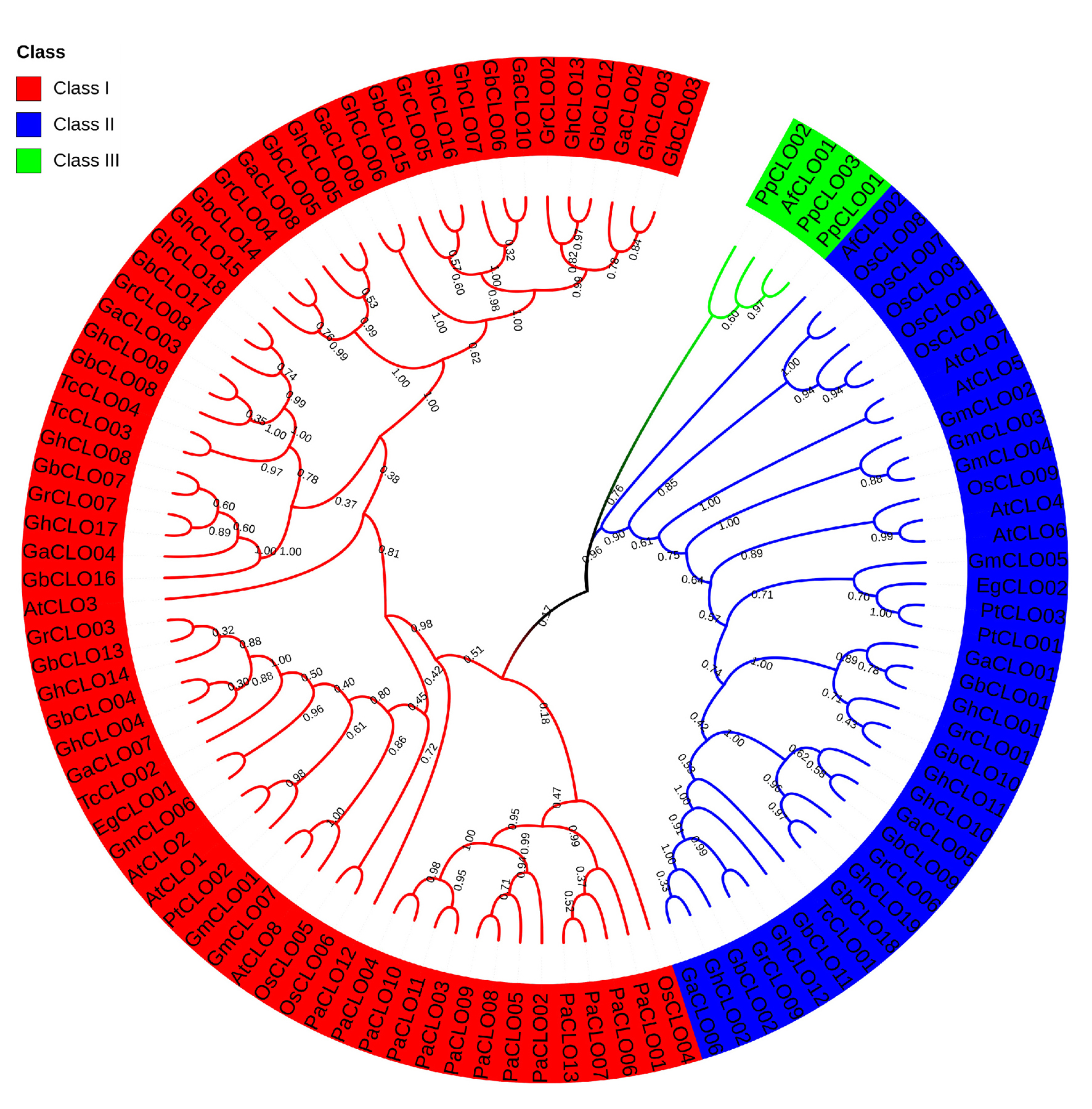

Supplement: Supplementary Figure 1 — Phylogenetic tree of the CLO proteins in 13 species. Three categories of CLO proteins are indicated using different colors by iTOL. [file Image_1.JPEG]

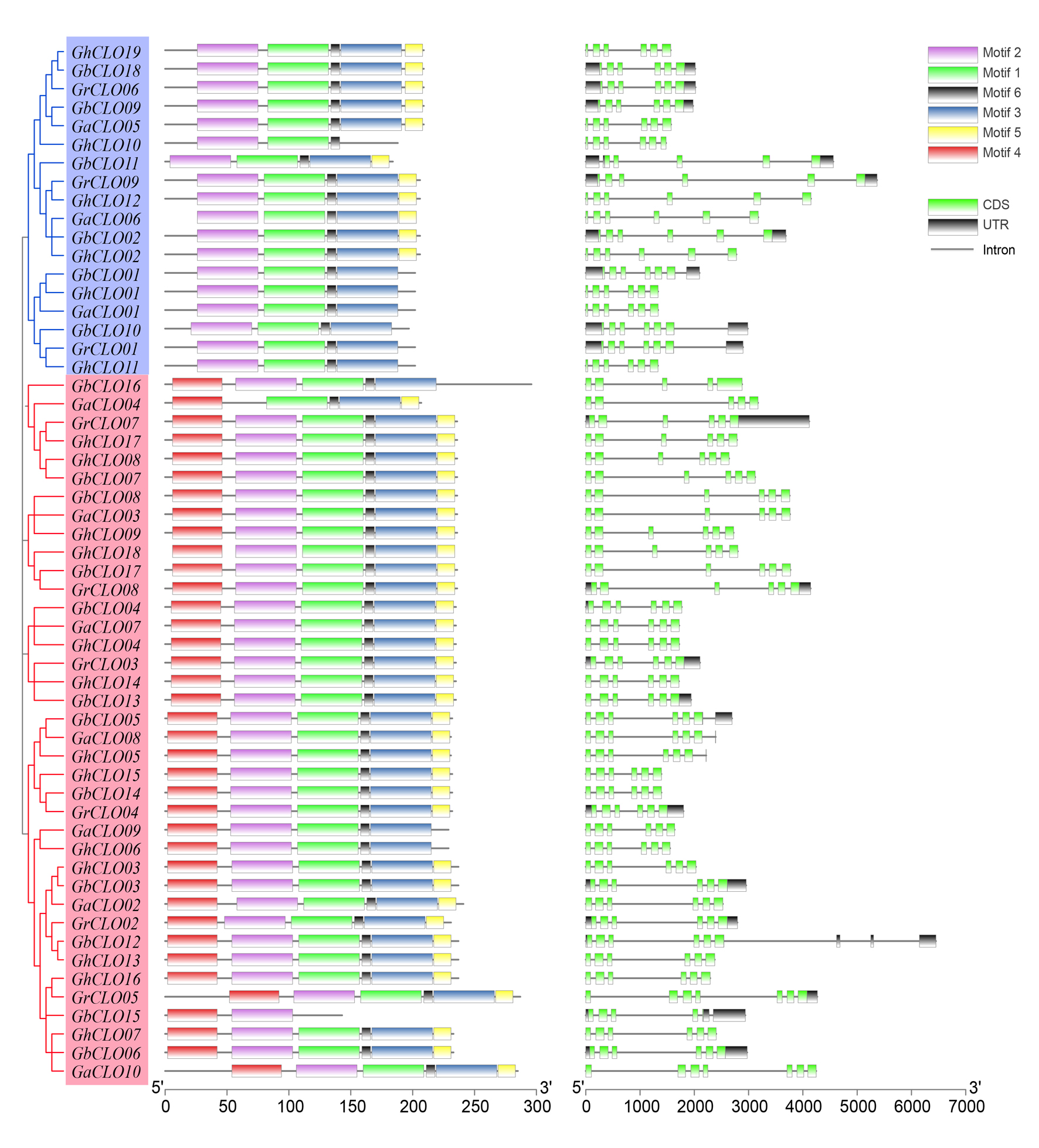

Supplement: Supplementary Figure 2 — Phylogenetically aligned conserved motif and gene structure analysis of CLO genes in four cotton species. [file Image_2.JPEG]

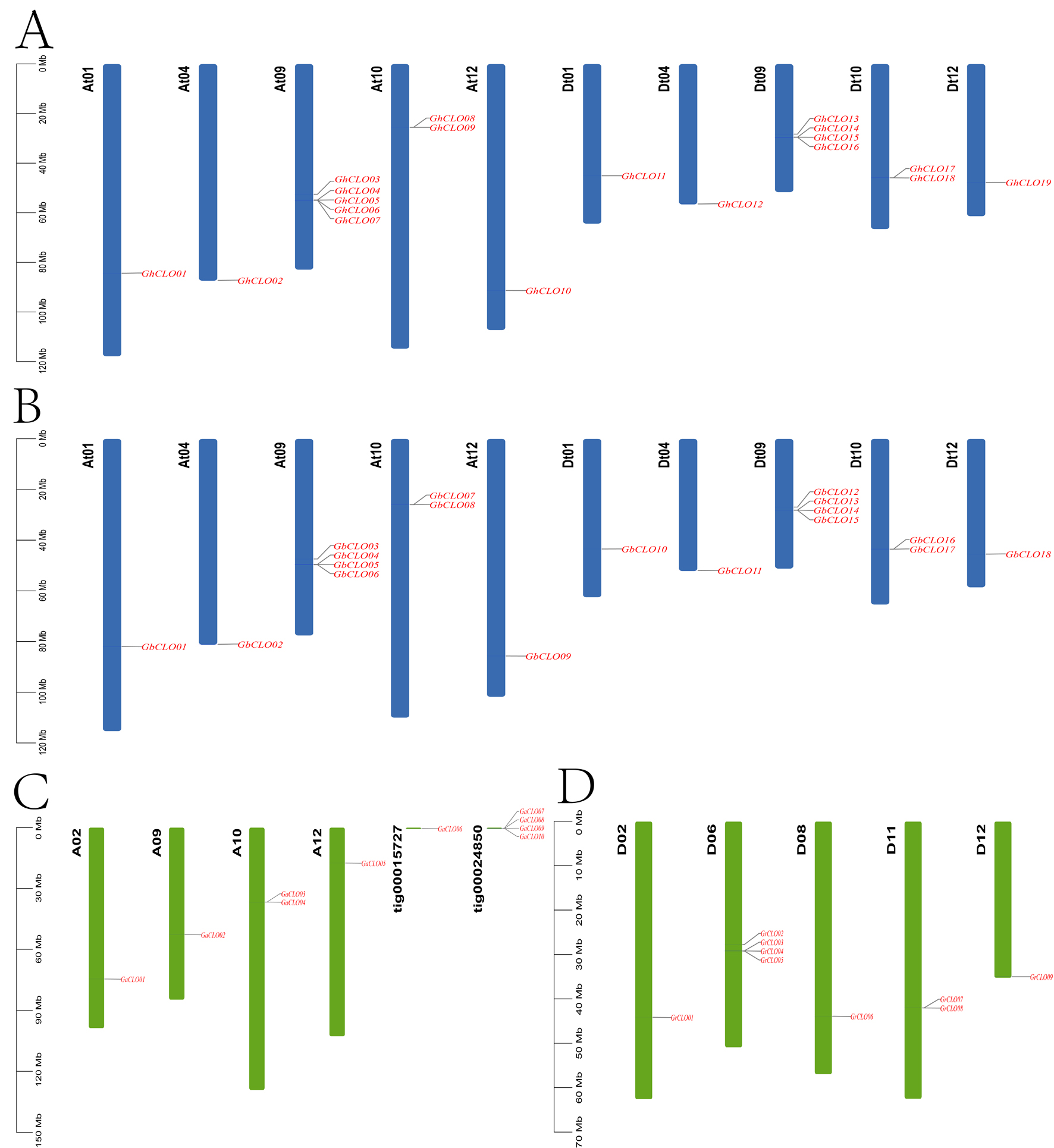

Supplement: Supplementary Figure 3 — Distribution of CLOs among the chromosomes of four cotton species. Panels (A–D) represent the chromosomal locations of CLO genes from Gossypium hirsutum (A), Gossypium barbadense (B), Gossypium arboreum (C), and Gossypium raimondii (D), respectively. The chromosome numbers are shown on the left side of each vertical bar. [file Image_3.JPEG]
